# The Risk of Mortality among Psoriatic Patients with Varying Severity: A Nationwide Population-Based Cohort Study in Taiwan

**DOI:** 10.3390/ijerph15122622

**Published:** 2018-11-22

**Authors:** Ying-Xiu Dai, Ming-Chun Hsu, Hsiao-Yun Hu, Yun-Ting Chang, Tzeng-Ji Chen, Chung-Pin Li, Chen-Yi Wu

**Affiliations:** 1 Department of Dermatology, Taipei Veterans General Hospital, Taipei 11217, Taiwan; daiinxiu@gmail.com (Y.-X.D.); miguel740118@hotmail.com (M.-C.H.); ytchang@vghtpe.gov.tw (Y.-T.C.); 2 School of Medicine, National Yang-Ming University, Taipei 11217, Taiwan; tjchen@vghtpe.gov.tw (T.-J.C.); cpli@vghtpe.gov.tw (C.-P.L.); 3 Institute of Public Health and Department of Public Health, National Yang-Ming University, Taipei 11217, Taiwan; hyhu@ym.edu.tw; 4 Department of Education and Research, Taipei City Hospital, Taipei 10341, Taiwan; 5 Department of Family Medicine, Taipei Veterans General Hospital, Taipei 11217, Taiwan; 6 Division of Gastroenterology and Hepatology, Department of Medicine, Taipei Veterans General Hospital, Taipei 11217, Taiwan

**Keywords:** psoriasis, psoriatic arthritis, mortality, National Health Insurance Research Database

## Abstract

*Background*: Previous studies showed conflicting results regarding the mortality risk in psoriasis patients with respect to disease severity and presence of psoriatic arthritis. This study aimed to determine the mortality risk in patients with mild and severe psoriasis and patients with psoriatic arthritis (PsA). *Methods*: A nationwide population-based cohort study was conducted based on data from the Taiwan National Health Insurance Research Database between 2002 and 2012. Incident psoriasis subjects were classified into two groups: psoriasis without arthritis and psoriasis with arthritis. Patients who had received systemic therapy and/or phototherapy were classified as having severe psoriasis; otherwise, patients were classified as having mild psoriasis. Control subjects without psoriasis were selected to match each psoriasis patient from the database within the same observational period. Cox proportional hazards analysis was used to compare the hazard ratio (HR) of time to death. *Results*: A total of 106,701 patients with psoriasis were included in this study. After controlling for demographics and comorbidities, psoriasis patients had a higher mortality risk compared with the control group (HR 1.41; 95% confidence interval (CI) 1.36 to 1.46). Compared with psoriasis alone, the mortality risk was not increased for PsA (HR = 1.01; 95% CI 0.93 to 1.10). Besides, severe psoriasis did not increase mortality risk compared with mild psoriasis (HR = 1.0; 95% CI 0.95 to 1.06). *Conclusions:* Patients with psoriasis had a higher mortality risk compared with control subjects, whereas psoriasis severity and presence of PsA had no impact on mortality risk in psoriasis patients.

## 1. Introduction

Psoriasis and psoriatic arthritis (PsA) are chronic disabling diseases that have a substantial negative impact on a patients’ quality of life, resulting in a great physical, emotional, and social burden [1]. An increasing trend in psoriasis and psoriatic arthritis prevalence has been observed in several countries [2,3,4,5,6,7], making them serious global problems.

Over the past decade there has been greater recognition of increased mortality associated with psoriasis [7,8,9,10], particularly due to cardiovascular disease [10,11]. Mortality studies have been carried out for psoriatic arthritis as well [8,10,12,13,14,15,16,17]. Most of the studies have been limited by their small sample size and selection bias in community- or hospital-based studies [12,14,15,16,17,18]. Besides, it is unclear whether disease severity and presence of PsA are associated with mortality risk in psoriasis patients. This study aimed to investigate the mortality risk in psoriasis patients with respect to the psoriasis severity and presence of PsA.

## 2. Materials and Methods

### 2.1. Data Source

A cohort study was conducted using data from the National Health Insurance Research Database (NHIRD), which covered over 99.9% of the nearly 23 million people in Taiwan between 2000 and 2012. The NHIRD database contains registration files and original reimbursement claims data, including demographic characteristics, outpatient and inpatient services, diagnostic codes, procedures performed, and details of prescriptions and comorbidities. The NHIRD has been widely used in epidemiological studies [19,20,21,22,23,24,25,26]. Previous studies have confirmed the high accuracy and validity of the NHIRD in recording psoriasis [10]. This study was performed in accordance with the Helsinki Declaration and was approved by the National Health Research Institutes and the Institutional Review Board of Taipei Veterans General Hospital (IRB: 2015-02-011CC).

### 2.2. Study Population and Study Design

We enrolled patients with a new diagnosis of psoriasis in the NHIRD between 1 January 2000, and 31 December 2001. Subjects with prior psoriasis diagnosis were excluded. The date of the first psoriasis diagnosis was defined as the index date, which was the start of follow-up for these patients. Subjects were considered to have psoriasis only if the diagnosis was made by dermatologists or rheumatologists and the condition occurred in an inpatient setting or required three or more outpatient visits. Psoriasis was identified by International Classification of Disease, Ninth Revision, Clinical Modification (ICD-9-CM) codes 696.0, 696.1, and 696.8. Patients were classified into groups with PsA, which were identified using ICD-9-CM code 696.0 during the same period; otherwise, patients were considered to have psoriasis without PsA (PsO). Psoriasis severity was classified as mild and severe. Severe psoriasis was defined as disease requiring systemic therapy and/or phototherapy. All other cases were classified as mild psoriasis.

### 2.3. Matched Controls without Psoriasis

For each patient with psoriasis, one matched control without psoriasis were randomly selected from the database within the same observational period. These subjects were matched for age and sex.

### 2.4. Outcome Measurement

Since the National Health Insurance is a mandatory universal health insurance program open to all Taiwanese residents, withdrawal from the insurance is almost always due to death. All study subjects were followed from the index date to withdrawal from the insurance or 31 December 2012, whichever date came first. Subjects with the condition mentioned were considered censored in the analysis.

### 2.5. Systemic Treatment

Systemic treatment was defined as any systemic anti-psoriatic therapy, including methotrexate, azathioprine, cyclosporine, systemic retinoids, mycophenolate mofetil, hydroxyurea, and biologic agents (i.e., etanercept, adalimumab, or ustekinumab).

### 2.6. Statistical Analysis

The demographic information of patients was compared by using χ^2^ tests for categorical variables and *t*-tests for continuous variables. Death rates per 1000 patient-years and 95% confidence intervals (CIs) were calculated according to psoriasis status. Cox proportional hazards analysis was used to compare the mortality risks. The model was adjusted for age, sex, socioeconomic status, residence (urban, suburban, or rural), and comorbidities (hypertension, diabetes mellitus, dyslipidemia, coronary artery disease, stroke, connective tissue disease, renal disease, chronic liver disease including cirrhosis and hepatitis, chronic obstructive pulmonary disease, and cancer). A two-sided *p* value of <0.05 was considered to represent a statistically significant difference. All data processing and statistical analyses were performed with Stata version 12 (Stata Corporation, College Station, TX, USA) and statistical analysis software (SAS) version 9.1 (SAS Institute, Cary, NC, USA).

## 3. Results

A total of 106,701 patients with incident psoriasis were identified. Among them, 8795 patients had PsA, and 22,542 patients had severe psoriasis. Baseline characteristics are shown in Table 1. The mean age was similar in patients with psoriasis (mean, standard deviation (SD) = 45.1 (18.7) years) and control subjects (mean (SD) = 45.1 (18.8) years). Compared with matched control subjects, psoriasis patients had a higher prevalence of multiple comorbidities, including hypertension, diabetes mellitus, dyslipidemia, coronary artery disease, stroke, connective tissue disease, renal disease, chronic liver disease, cirrhosis and hepatitis, chronic obstructive pulmonary disease, and cancer.

As shown in Table 2, PsA patients (mean (SD) = 44.5 (16.4) years) were younger on average than PsO patients (mean (SD) = 45.2 (18.9) years). Patients with PsA were more likely to have a comorbidity including dyslipidemia (26.3% vs. 24.7%, *p* < 0.001), connective tissue disease (20.8% vs. 4.9%, *p* < 0.001), renal disease (8.1% vs. 7.4%, *p* < 0.001), and chronic liver disease, cirrhosis and hepatitis (26.5% vs. 21.2%, *p* < 0.001) and were more like to have received systemic treatment (45% vs. 10.6%, *p* < 0.001) or phototherapy (23.4% vs. 11.9%, *p* < 0.001) than those with PsO. On the contrary, the incidence of coronary artery disease (15.3% vs. 14.1%, *p* = 0.003), stroke (10.8% vs. 9.1%, *p* < 0.001), chronic obstructive pulmonary disease (19.5% vs. 17.3%, *p* < 0.001), and cancer (7.1% vs. 6.4%, *p* = 0.02) was higher in PsO patients than in PsA patients.

A shown in Table 3, the mean age was similar in patients with mild psoriasis (mean (SD) = 45.1 (19.0) years) and severe psoriasis (mean (SD) = 5.0 (17.6) years). Compared with patients with mild psoriasis, those with severe psoriasis were more likely to have comorbidities of hypertension (36.7% vs. 33.1%, *p* < 0.001), diabetes mellitus (20.6% vs. 18%, *p* < 0.001), dyslipidemia (27.3% vs. 24.1%, *p* < 0.001), connective tissue disease (11.5% vs. 4.8%, *p* < 0.001), renal disease (9% vs. 7.1%, *p* < 0.001), chronic liver disease including cirrhosis and hepatitis (25.9% vs. 20.5%, *p* < 0.001), and cancer (7.8% vs. 6.8%, *p* < 0.001).

During an average of 5.2 (SD 3.1) years of follow-up, the mortality rate was 15.8 per 1000 person-years in psoriasis patients. Compared with control subjects, psoriasis patients had a significantly higher risk of mortality (incidence rate ratio = 1.46; 95% CI 1.41 to 1.51) after multivariable adjustment (hazard ratio (HR) = 1.41; 95% CI 1.36 to 1.46) (Table 4). Both mild and severe psoriasis and presence and absence of PsA are associated with an increased mortality risk. Patients with PsA did not have a higher mortality risk compared with those with PsO (HR = 1.01; 95% CI 0.93 to 1.10). There was no significant difference in mortality risk between patients with severe psoriasis and those with mild psoriasis (HR = 1.00; 95% CI 0.95 to 1.06).

## 4. Discussion

In this nationwide population-based study, we found that psoriasis patients with or without arthritis and with mild or severe psoriasis have a significantly increased risk of mortality compared with the control subjects. Our findings are in agreement with those of prior studies [8,10,27,28].

In the studies by Wong et al. [15], Ali et al. [16], and Mok et al. [18], PsA patients had a 1.62-fold, a 1.36-fold, and 1.59-fold increased mortality risk, respectively. However, Wilson et al. [17] and Buckley et al. [12] did not find a significant increase in mortality in PsA patients compared with the general population. Two recent population-based cohort studies showed conflicting results. Ogdie et al. [8] found that the death rate in 8706 PsA subjects did not differ from that of the general population, whereas Lee et al. [10] found increased mortality in 9572 PsA patients.

Several studies investigated the mortality risk in psoriasis patients with respect to disease severity [8,10,11,26,27,28]. Among these studies, four suggested an increased mortality in both patients with mild and severe psoriasis compared with the general population [8,10,27,28]; one reported a significant higher risk of mortality in patients with severe psoriasis [11]. Another study identified that severe but not mild psoriasis as associated with an increased risk of death [29]. Growing evidence suggests a link between psoriasis and other comorbidities. Psoriasis patients are more likely to have malignancy, cardiovascular disease, metabolic syndrome, obesity, and diabetes mellitus, particularly in those with PsA and severe skin disease [27,28,30,31,32], which could explain the higher mortality risk observed in psoriasis patients. However, in our study, the psoriasis severity and presence of PsA had no impact on the overall mortality risk in psoriasis patients. The non-significant mortality difference between PsO patients and PsA patients could be explained by higher prevalence of cardiovascular disease and malignancy in PsO patients. Compared with the studies of Ogdie et al. [8] and Lee et al. [10] which enrolled only adult patients (age ≥18 years), our study included patients aged under 18 years. Since younger patients are less vulnerable to morbidity and mortality, the inclusion of these patients in our cohort might partially explain the nonsignificant effects of psoriasis severity on mortality risk in psoriasis patients. However, further studies are needed to determine the underlying explanations for our findings.

The strengths of our study include the large sample size, a population-based cohort study design, reliable psoriasis diagnosis made by dermatologists and rheumatologists, and a more comprehensive control of potential confounding factors. However, potential limitations of our study should be considered. First, since the NHIRD data did not include clinical assessments, it is not possible to classify psoriasis severity based on clinical measures such as the Physician Global Assessment and Psoriasis Assessment Severity Index. The use of treatment patterns as a marker for psoriasis severity may introduce misclassification bias. It is possible that some untreated patients with severe psoriasis were misclassified as having mild psoriasis. Nevertheless, previous studies have affirmed the reliability and validity of using these methods for grouping severe psoriasis [33,34,35,36]. Second, there could be a “healthy user” effect in that patients with severe psoriasis need to be healthy enough to be prescribed the therapies, which would result in an underestimate of mortality risk. Third, instead of confirming death with the death certificate data, we identified death using the subjects’ withdrawal from insurance. Withdrawal from insurance could be due to a renunciation of citizenship. However, this method has been validated in a previous study [37]. Fourth, the information regarding causes of mortality is lacking in our database, therefore, precluding us from further analysis in this study. Finally, the Taiwanese population is predominantly of Chinese descent and caution is needed in extrapolating the results to other ethnic groups.

## 5. Conclusions

In summary, our study demonstrated that there was a significantly increased risk of mortality in psoriasis patients. Both mild and severe psoriasis and presence and absence of PsA were associated with an increased mortality risk, whereas the psoriasis severity and presence of PsA had no impact on mortality risk in psoriasis patients. These findings urge physicians to provide comprehensive health assessments and preventive health practices to psoriasis patients even with mild skin disease and no PsA.

## Figures and Tables

**Table 1:** Baseline characteristics of study sample.

Variables	Patients with Psoriasis (*n =* 106,701)		Controls (*n =* 106,701)		*p*
	*n*	%	*n*	%	
Total number of patients with psoriasis	106,701				
Psoriatic arthritis	8795	8.2			
Psoriasis with phototherapy	13,742	12.9			
Psoriasis with systemic therapy	14,314	13.4			
Psoriasis with phototherapy or systemic therapy	22,542	21.1			
Sex					
Male	62,766	58.8	62,766	58.8	1.00
Female	43,935	41.2	43,935	41.2	
Socioeconomic status					
Low	55,814	52.3	53,027	49.7	<0.001
Medium	31,281	29.3	32,003	30	
High	19,606	18.4	21,671	20.3	
Residence					
Urban	62,902	59	65,208	61.1	<0.001
Suburban	34,364	32.2	31,968	30	
Rural	9435	8.8	9525	8.9	
Comorbidity					
Hypertension	36,156	33.9	23,916	22.4	<0.001
Diabetes mellitus	19,769	18.5	9822	9.2	<0.001
Dyslipidemia	26,474	24.6	15,706	14.7	<0.001
Coronary artery disease	16,224	15.2	9379	8.8	<0.001
Stroke	11,412	10.7	7043	6.6	<0.001
Connective tissue disease	6599	6.2	2220	2.1	<0.001
Renal disease	7993	7.5	4044	3.8	<0.001
Chronic liver disease, cirrhosis and hepatitis	23,107	21.7	14,143	13.3	<0.001
Chronic obstructive pulmonary disease	20,599	19.3	13,648	12.8	<0.001
Cancer	7511	7	5384	5.1	<0.001

**Table 2:** Characteristics of psoriatic patients according to existence of arthritis.

Variables	Psoriatic Patients				*p*
	without Arthritis		with Arthritis		
	*n*	%	*n*	%	
Total number of patients	97,906	91.8	8795	8.2	
Sex					
Male	57,532	58.8	5234	59.5	0.17
Female	40,374	41.2	3561	40.5	
Comorbidity					
Hypertension	33,140	33.9	3016	34.3	0.40
Diabetes mellitus	18,158	18.6	1611	18.3	0.60
Dyslipidemia	24,161	24.7	2313	26.3	<0.001
Coronary artery disease	14,982	15.3	1242	14.1	0.003
Stroke	10,616	10.8	796	9.1	<0.001
Connective tissue disease	4771	4.9	1828	20.8	<0.001
Renal disease	7285	7.4	708	8.1	0.04
Chronic liver diseases, cirrhosis, and hepatitis	20,776	21.2	2331	26.5	<0.001
Chronic obstructive pulmonary disease	19,080	19.5	1519	17.3	<0.001
Cancer	6947	7.1	564	6.4	0.02
Received phototherapy	11,684	11.9	2058	23.4	<0.001
Received systemic treatment	10,359	10.6	3955	45	<0.001

**Table 3:** Characteristics of psoriatic patients according to disease severity.

Variables	Psoriatic patients				*p*
	Mild		Severe		
	*n*	%	*n*	%	
Total number of patients	84,159	78.9	22,542	21.1	
Sex					
Male	47,978	57	14,788	65.6	<0.001
Female	36,181	43	7754	34.4	
Comorbidity					
Hypertension	27,873	33.1	8283	36.7	<0.001
Diabetes mellitus	15,134	18	4635	20.6	<0.001
Dyslipidemia	20,315	24.1	6159	27.3	<0.001
Coronary artery disease	12,675	15.1	3549	15.7	0.01
Stroke	9103	10.8	2309	10.2	0.01
Connective tissue disease	4000	4.8	2599	11.5	<0.001
Renal disease	5970	7.1	2023	9	<0.001
Chronic liver diseases, cirrhosis, and hepatitis	17,274	20.5	5833	25.9	<0.001
Chronic obstructive pulmonary disease	16,289	19.4	4310	19.1	0.43
Cancer	5749	6.8	1762	7.8	<0.001

**Table 4:** Risk of mortality in psoriatic patients.

	Number of Participants	Number of Deaths	Number of Deaths per 1000 Person-Years	Incidence Rate Ratio (95% CI)	Age- and Sex-Adjusted HR (95% CI)	Multivariable-Adjusted HR * (95% CI)
Control	106,701	5998	10.8	1	1	1
Total number of psoriatic patients	106,701	8626	15.8	1.46 (1.41–1.51)	1.48 (1.44–1.53)	1.41 (1.36–1.46)
Psoriatic patients without arthritis	97,906	8053	16	1.49 (1.44–1.54)	1.48 (1.43–1.53)	1.41 (1.36–1.46)
Psoriatic patients with arthritis	8795	573	12.6	1.17 (1.07–1.27)	1.56 (1.43–1.70)	1.52 (1.39–1.66)
Mild psoriatic patients	84,159	6780	16.1	1.50 (1.44–1.55)	1.48 (1.43–1.53)	1.41 (1.36–1.46)
Severe psoriatic patients	22,542	1846	14.5	1.34 (1.27–1.41)	1.51 (1.43–1.59)	1.43 (1.35–1.51)
Psoriatic patients without arthritis	97,906	8053	16	1	1	1
Psoriatic patients with arthritis	8795	573	12.6	0.79 (0.72–0.86)	0.99 (0.91–1.08)	1.01 (0.93–1.10)
Mild psoriatic patients	84,159	6780	16.1	1	1	1
Severe psoriatic patients	22,542	1846	14.5	0.90 (0.85–0.95)	0.99 (0.94–1.05)	1.00 (0.95–1.06)

* Adjusted for age, sex, socioeconomic status, residence, hypertension, diabetes mellitus, dyslipidemia, coronary artery disease, stroke, connective tissue disease, renal diseases, chronic liver diseases and cirrhosis and hepatitis, chronic obstructive pulmonary disease, and cancer. CI, confidence interval; HR, hazard ratio.
